# Improving Treatment Response Prediction for Chemoradiation Therapy of Pancreatic Cancer Using a Combination of Delta-Radiomics and the Clinical Biomarker CA19-9

**DOI:** 10.3389/fonc.2019.01464

**Published:** 2020-01-08

**Authors:** Haidy Nasief, William Hall, Cheng Zheng, Susan Tsai, Liang Wang, Beth Erickson, X. Allen Li

**Affiliations:** ^1^Department of Radiation Oncology, Medical College of Wisconsin, Milwaukee, WI, United States; ^2^Joseph J. Zilber School of Public Health, University of Wisconsin Milwaukee, Milwaukee, WI, United States; ^3^Department of Surgery, Medical College of Wisconsin, Milwaukee, WI, United States; ^4^Department of Pathology, Medical College of Wisconsin, Milwaukee, WI, United States

**Keywords:** pancreatic cancer, survival, CA19-9, biomarkers, chemo-radiation therapy, response assessment

## Abstract

Recently we showed that delta radiomics features (DRF) from daily CT-guided chemoradiation therapy (CRT) is associated with early prediction of treatment response for pancreatic cancer. CA19-9 is a widely used clinical biomarker for pancreatic cancer. The purpose of this work is to investigate if the predictive power of such biomarkers (DRF or CA19-9) can improve by combining both biomarkers. Daily non-contrast CTs acquired during routine CT-guided neoadjuvant CRT for 24 patients (672 datasets, in 28 daily fractions), along with their CA19-9, pathology reports and follow-up data were analyzed. The pancreatic head was segmented on each daily CT and radiomic features were extracted from the segmented regions. The time between the end of treatment and last follow-up was used to build a survival model. Patients were divided into two groups based on their pathological response. Spearman correlations were used to find the DRFs correlated to CA19-9. A regression model was built to examine the effect of combining CA19-9 and DRFs on response prediction. C-index was used to measure model effectiveness. The effect of a decrease in CA19-9 levels during treatment vs. failure of CA19-9 levels to normalize on survival was examined. Univariate- and multivariate Cox-regression analysis were performed to determine the effect of combining CA19-9 and DRFs on survival correlations. Spearman correlation showed that CA19-9 is correlated to DRFs (Entropy, cluster tendency and coarseness). An Increase in CA19-9 levels during treatment were correlated to a bad response, while a decline was correlated to a good response. Incorporating CA19-9 with DRFs increased the c-index from 0.57 to 0.87 indicating a stronger model. The univariate analysis showed that patients with decreasing CA19-9 had an improved median survival (68 months) compared to those with increasing levels (33 months). The 5-years survival was improved for the decreasing CA19-9 group (55%) compared to the increasing group (30%). The Cox-multivariate analysis showed that treatment related decrease in CA19-9 levels (*p* = 0.031) and DRFs (*p* = 0.001) were predictors of survival. The hazard-ratio was reduced from 0.73, *p* = 0.032 using CA19-9 only to 0.58, *p* = 0.028 combining DRFs with CA19-9. DRFs-CA19-9 combination has the potential to increasing the possibility for response-based treatment adaptation.

## Introduction

Pancreatic cancer (PC) is a devastating malignancy and one of the leading causes of cancer death in the United States. The American cancer society estimates that about 56,770 people will be diagnosed with PC in 2019 (1). Precise oncologic profiling in early stages is very critical to improve prognosis. Although advances in cancer care have substantially improved outcomes for particular tumor sites over the past years, little change has been seen in the pancreatic cancer patient outcomes. Surgical resection is considered a prerequisite for curing pancreatic cancer and achieving long term survival. However, <10% of patients with pancreatic tumors have resectable tumors at the time of presentation due to the nature of the tumor and its relationship to the surrounding vascularity (2). Tumor degree of abutment to the superior mesenteric artery (SMA) and/or celiac artery and the size of the tumor is a major determinant of resectability. Based on our institutional recommendations, tumor with <180° abutment in the SMA or celiac artery or with short segment abutment/encasement without extension to celiac artery or hepatic artery bifurcation or >50% narrowing in the tumor-vein anatomy are considered borderline resectable. Neoadjuvant therapy with chemo-radiation usually precedes surgery to downstage the tumor from being borderline resectable to resectable (3).

Clinically, Carbohydrate antigen (CA19-9) has been extensively studied and widely accepted as a tumor biomarker for PC. For instance, Waraya et al. performed a multivariate analysis of factors predicting survival for patients undergoing surgical resection and reported that low preoperative CA19-9 serum levels and positive peripancreatic margins independently predict overall survival (4). Turrini et al. reported a median survival of 22 months for the group with a low CA19-9 compared to 12.7 months for the elevated CA19-9 group (5). Kondo et al. reported a median survival of 57 months for the group with a low CA19-9 vs. 30 months for those with elevated levels (6). These studies have demonstrated that a treatment related decline in CA19-9 serum levels is associated with prolonged survival and is an independent predictor of overall survival, and that an elevated CA19-9 serum level is associated with poor prognosis. However, CA19-9 if used alone, suffers from low sensitivity because other factors that are not tumor related can elevate the CA19-9 levels, Kim et al. demonstrated that preoperative serum CA19-9 and CEA levels can be used for the prediction of resectability with an accuracy of 27.1% if either tumor marker is used and 40.6% if both are used (7). Steinberg, Duraker et al., and Kim et al. found that interpretation of CA19-9 can be falsely elevated by the presence of benign conditions, such as ovarian cysts, heart failure, Hashimoto's thyroiditis, rheumatoid arthritis, diverticulitis, or biliary obstruction with elevation of serum bilirubin (8–10). The presence of such conditions can increase the false positive rate. An option to address this problem is to associate CA19-9 with another biomarker that can confirm its tumor related elevation and hence, increase the sensitivity and reduce the false positive rates.

CT is a non-invasive imaging modality that is used to monitor oncologic changes and/or to assess treatment response for cancer treatment. Radiomics is the field that converts these medical images into quantitative data. CT-derived radiomic texture features have shown promising prognostic value in a variety of cancer treatments. For instance, Hou et al. performed radiomic analysis using contrast-enhanced CT and found that the identified radiomic features have the potential to predict treatment response in esophageal carcinoma with an AUC of 0.97 (11). Coroller et al. showed that CT-based radiomics can be developed as a prognostic biomarker to predict distant metastasis in lung cancer (12). Eilaghi et al. reported that CT texture features of the dissimilarity and normalized inverse difference were associated with overall survival for PC (13).

Delta radiomics is a form of radiomics and is introduced to assess the relative net change of radiomic features in a set of longitudinal images. Delta radiomic feature (DRF) can be derived from a variety of radiomic metrics in conjunction with clinical outcomes. The presence of a trend in DRF during treatment may indicate a good or poor response to the treatment. For instance, Chen et al. showed that the first-order CT DRFs could potentially be used for early assessment of treatment response during chemoradiation therapy (CRT) for PC (14). Al-Kadi and Watson showed that fractal texture changes in time-sequenced contrast-enhanced CT images could potentially impact the clinical decision for choosing the appropriate treatment plan for aggressive and non-aggressive malignant tumors (15). Fave et al. used DRFs to create a model for survival and distant metastases in lung and found that adding radiomic features improved the prognostic power of their model (16). Our previous analysis showed that DRFs including kurtosis, NESTD, and coarseness can predict treatment response for resectable pancreatic cancer with AUC of 0.94 (17–19). Although DRFs have been associated with several clinical endpoints in a variety of applications, the complex relationships of radiomics and clinical factors are largely unknown. Particularly for CRT of PC, the predictive power of DRF or CA19-9 for response assessment and/or survival is limited if used alone. The purpose of this work is to investigate if the predictive power and prognostic value can be improved by combining both DRF and CA19-9. We will analyze the clinical data collected for both resectable and borderline resectable PC patients.

## Materials and Methods

In this retrospective, Medical college of Wisconsin IRB approved HIPPA compliant study (consent forms are waived for retrospective study), daily non-contrast CTs acquired during routine CT-guided CRT for 24 patients (standard imaging protocol, 28 daily CTs for each patient, a total of 672 CT sets), along with their CA19-9 tests, post-CRT pathology reports and follow up data were analyzed. Inclusion criteria included (1) patients who have multiple CA19-9 test results before, after and within the course of treatment (enough for assessing weekly changes), (2) no other apparent non-tumor related factors that are known to elevate CA19-9 (i.e., no liver diseases, no gallstones, all patients had biliary stent to avoid inflammation, no pancreatitis and no jaundice), (3) patients with PT3N0M0R0 resectable or borderline resectable pancreatic head tumors (equal number of resectable and borderline resectable tumors were included. To reduce variations in the data, patients included in the study underwent the same CRT scheme and treatment protocol. All patients were treated with pre-operative chemotherapy with Folfirinox and concurrent CRT with gemcitabine in 28 daily fractions followed by surgery during the period between 2012 and 2017. The mean age for the patients included in the study was 65 years old at the start of treatment, with 70% being male and 30% female.

All patients underwent surgery after CRT and the pathological treatment response was assessed from the serially sectioned formalin-fixed surgical specimen the area of tumor and surrounding fibrosis in the pancreas was submitted for microscopic examination. Hematoxylin and eosin sections were prepared, and treatment effect was evaluated. A modified Ryan Scheme for tumor regression score recommended by the College of American Pathologists was used to evaluate treatment effect as follows: Grade 0 (G0): no viable cancer cells (complete response), Grade 1 (G1): single cells or small groups of residual cancer (near complete response), Grade 2 (G2): residual cancer with evident tumor regression, but more than single cells or rare small groups of cancer cells (partial response), and Grade 3 (G3): extensive residual cancer with no evident of tumor regression (poor or no response) (20). Among the 24 patients, there were 12 patients with good pathological response (G1 and G2) and 12 with bad pathological response (G3). These pathological data were used for the response correlation analysis.

**Figure 1:**
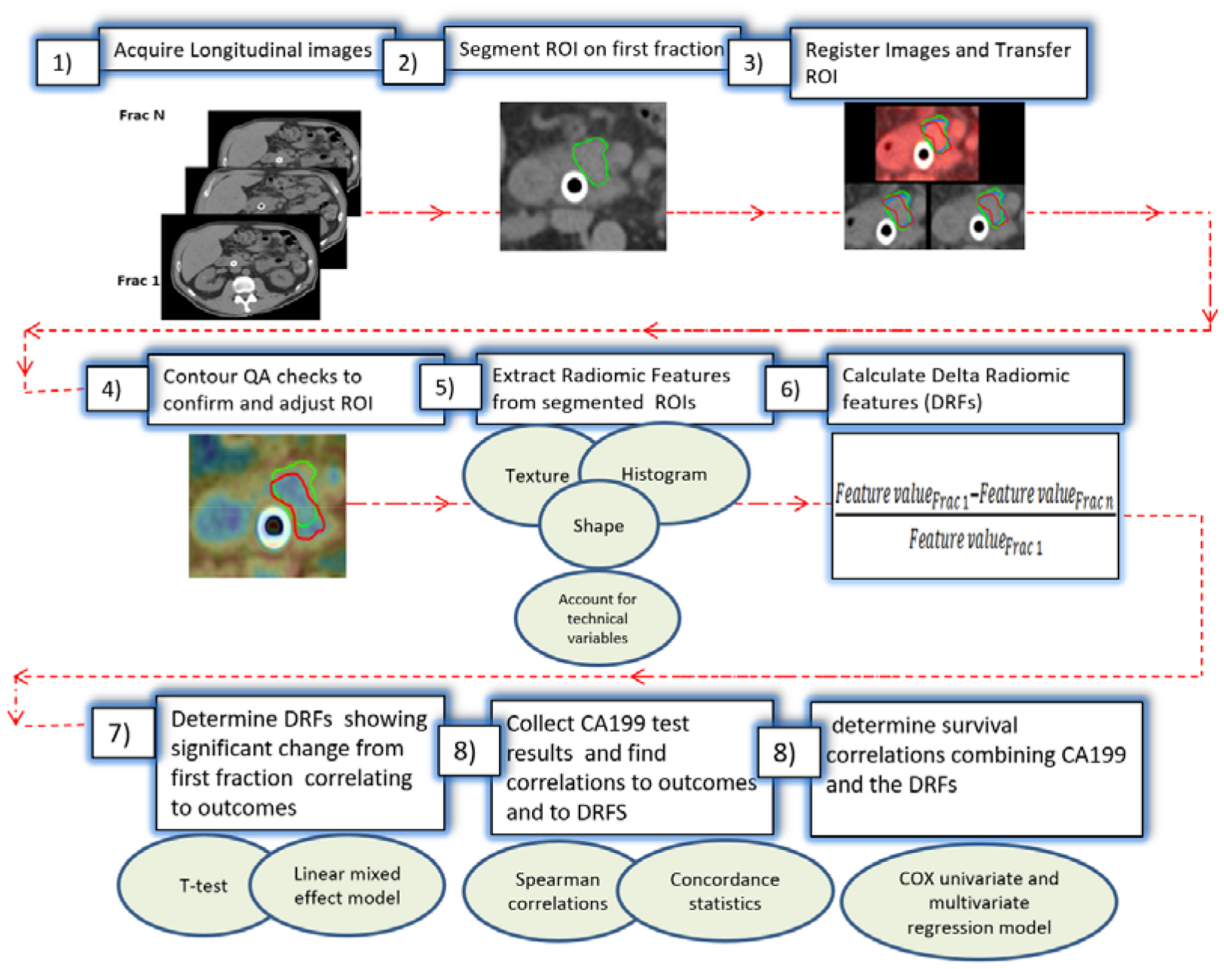
Work flow of combining the delta-radiomic and CA19-9.

To avoid technical variations, patients included in this study were all scanned using the same CT scanner (Definition AS, Siemens), with a standard abdominal protocol consisting of the following parameters: 120 kVp tube voltage, 252 mA tube current, 0.5 s, 1.2 mm focal spot, and standard filtered back-projection (FBP) algorithm with B30f kernels. All analyzed CTs were reconstructed in a 512 × 512 × Z (slices) voxels with resolution 0.98 × 0.98 × 3 mm. The daily CTs were acquired with respiratory gating, reducing the motion to below 3 mm (the residual motion in the gating window) during the CT acquisition (21). A delta radiomic process was performed to extract radiomic features from the regions of interest (ROIs) as shown in Figure 1. The process starts with acquiring the longitudinal CT images from the daily CTs. Daily CTs were registered rigidly with each other with manual adjustment, if necessary, to achieve the best local matching between the two CT sets. For each case, the pancreatic head (excluding the stent) was segmented delineated on the contrast-enhanced simulation CT and MRI and was populated to the CT of the first fraction, then to other daily CTs based on rigid image registration. The segmented ROIs were inspected and edited if necessary, by experienced researchers using MIM software and verified independently by other experienced researchers and oncologists to ensure consistency. Contour QA checks were also applied to reduce the interobserver contouring variability using our newly developed NESTD map (defined as the normalized entropy to standard deviation difference on a voxel by voxel basis) to enhance boundary detection and to adjust the contours if tissues other than the pancreatic head is included in the ROIs.

Over 1,300 radiomic features were extracted using IBEX software (22) from the segmented regions. Features extracted included intensity-based histogram, gray level co-occurrence matrix, neighbor gray tone difference matrix, gray level run length matrix, Intensity histogram Gaussian fit, and Shape-based features, and our newly developed NESTD feature (defined as the normalized entropy to standard deviation difference on a voxel by voxel basis) (23–32). Since relatively high numbers of extracted radiomic features compared to the sample size can reduce the statistical power and increase the overfitting probability, a Spearman rank-order correlation coefficient was used to rule out low-rank redundant features (*r*_*s*_ > 0.9). Also, to reduce directional dependence of texture features, direction-specific matrices are summed and averaged to create a final global matrix. This resulted in 73 DRFs that could be used for delta-radiomic analysis (17–19). In this study, patients were divided into good- and poor-response groups based on their treatment responses. Changes in each of the 73 DRFs were calculated as the relative net change of the radiomic feature of interest from the first fraction as follows (19):

(1) $$\begin{array}{l} DRFn=\frac{{Feature\;value}_{Frac\;1}-{Feature\;value}_{Frac\;n\;}}{{Feature\;value}_{Frac\;1}},\\ \begin{array}{r} \;where\;n=2:28 \end{array} \end{array}$$

A metric trend was established using a linear regression model to find the best fit for each of the DRFs features vs. response and subsequently determine those with potential trends. Features were also evaluated to determine if they changed during the treatment using a *t*-test and were tested for whether related to response by fitting linear mixed effects models using R® software for the change of DRFs as a function of response with two random effects to account for patient- and fraction-dependent variations as follows (19):

(2) ⌈model = lme(DRF ~ Response +  (1|Patient) +(1|Fraction) ⌉

The *p*-value of the log likelihood ratio for each model was calculated. The features that passed both the linear mixed effect model and the *t*-test (*p* < *0.05*) were selected to be used for subsequent analysis.

Unlike DRFs, the CA19-9 is a blood test and thus cannot be collected for each fraction for each patient. Patients selected in this study, had CA19-9 test results available at the start, the end of the treatment and at additional time points within the treatment. CA19-9 test results were obtained from all available fractions for each patient. To obtain the weekly changes in CA19-9 test results and be able to combine data from different patients given the wide range CA19-9 values can span, the values were normalized in a similar fashion to the DRFs. The relative net change of CA19-9 was obtained by comparing the CA19-9 value for each available fraction with respect to a starting point of the blood sample collected within the week post-simulation and prior to radiation. The first week data were obtained from patients with available CA19-9 blood test collected within the first week of treatment and the last week data were obtained from all available CA19-9 for any available fractions within the last week of treatment or within 2 days after last day of treatment and prior to surgery. The rest of the weeks contained data from the patients with available CA19-9 for any fraction within that week (for instance if one patient has CA19-9 data collected at a time point corresponding to Frac 17 and the other have CA19-9 corresponding to Frac 18, these data were combined in a boxplot representing this week). All available CA19-9 test results from all patients were used to generate weekly boxplots and *t*-test was used to determine significant changes. Spearman correlation coefficients were used to find which of the significant DRFs are highly correlated to CA19-9 for survival correlations. The time between the end of the treatment and the presence of an endpoint of death or presence of metastasis were used to build the survival model. Patients who did not reach an endpoint were censored at their last follow-up.

A regression model was built to examine the effect of combining changes in CA19-9 and DRFs on response correlations. A concordance statistic was used to reflect the ability of the prognostic model to correctly identify treatment response. A value below 0.5 indicates a very poor model, 0.5 means that the model is no better than predicting an outcome than random chance, 0.7–0.8 indicate a good model, over 0.8 indicate a strong model, and 1 means that the model perfectly predicts tumor response. A Cox proportional hazard regression model was built to examine the effect of combining CA19-9 and DRFs on survival prediction. Hazard ratio was used to determine the prognostic value of combining CA19-9 and DRFs on progression free survival.

## Results

**Figure 2:**
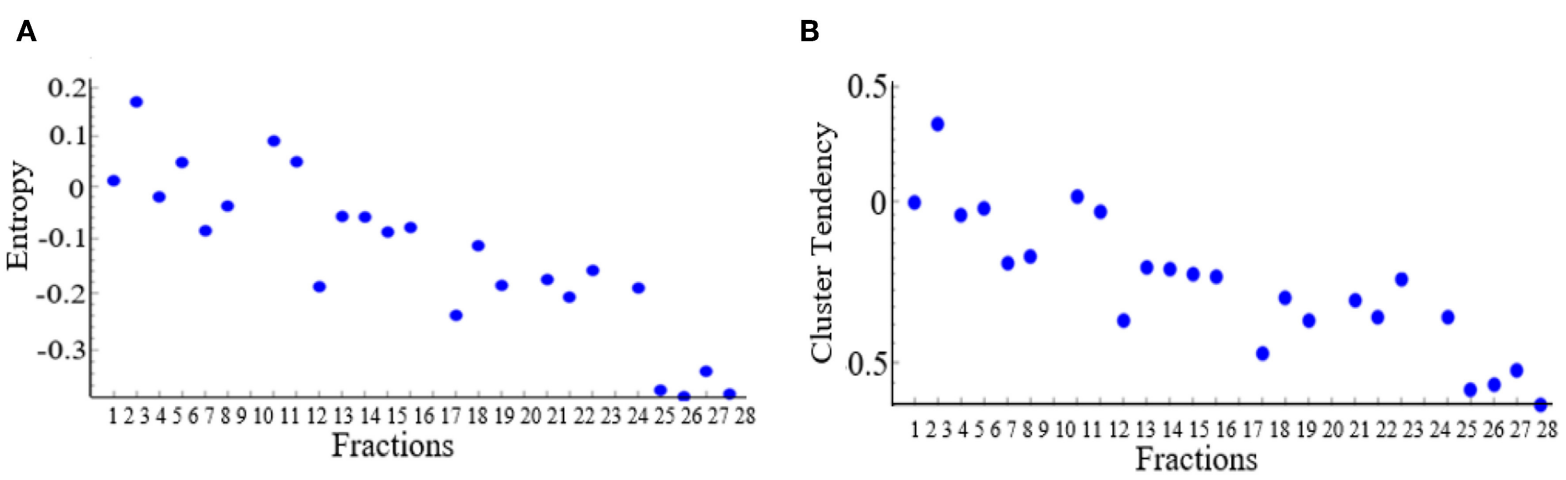
Changes of selected delta radiomic features [**(A)** Entropy and **(B)** cluster tendency] as a function of increasing treatment fraction. Each dot represents the average value of all good responders for a treatment time point.

The results showed that 13 DRFs (complexity, cluster tendency, coarseness, information measure, contrast, entropy, inverse variance, gray level non-uniformity, mean, IDN, kurtosis, skewness, and NESTD) showed a trend correlating to pathological treatment response and this trend was opposite between good and bad response groups. Figure 2 shows examples of DRFs (Entropy and cluster tendency) showing a decreasing trend with increasing treatment fractions (radiation dose increase) for all good responders.

**Figure 3:**
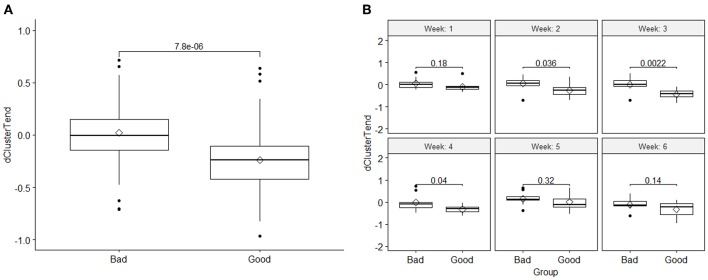
**(A)** Comparison of the DRF (cluster tendency) values between the two response groups and the *t*-test *p*-value, and **(B)** a *t*-test comparison of weekly DRFs between the good and bad response groups for the DRF (cluster tendency). The boxplots show the median and interquartile range for each response group and the diamond data point in the middle represents the mean of the group.

Based on the *t*-test and the linear mixed effect analysis, these DRFs passed both tests (*p* < 0.05) indicating significant difference between the two response groups when combining all fractional values for all patients within each response groups. This significant difference was more pronounced between the second to fourth week of treatment. Figure 3 shows an example of boxplots and corresponding *t*-test *p*-value for (A) DRF values of all fractions for cluster tendency in each group, showing significant changes between the good and bad response groups, and (B) a *t*-test comparison of weekly values (combining all fractional values for all patients within each week) between the good and bad response groups for the DRF (cluster tendency). These data indicate the significant changes of the selected DRFs from the first fraction to the subsequent weeks during the treatment. The presented boxplots show the median and interquartile range for each response group and the diamond data point in the middle represents the mean of the group.

**Figure 4:**
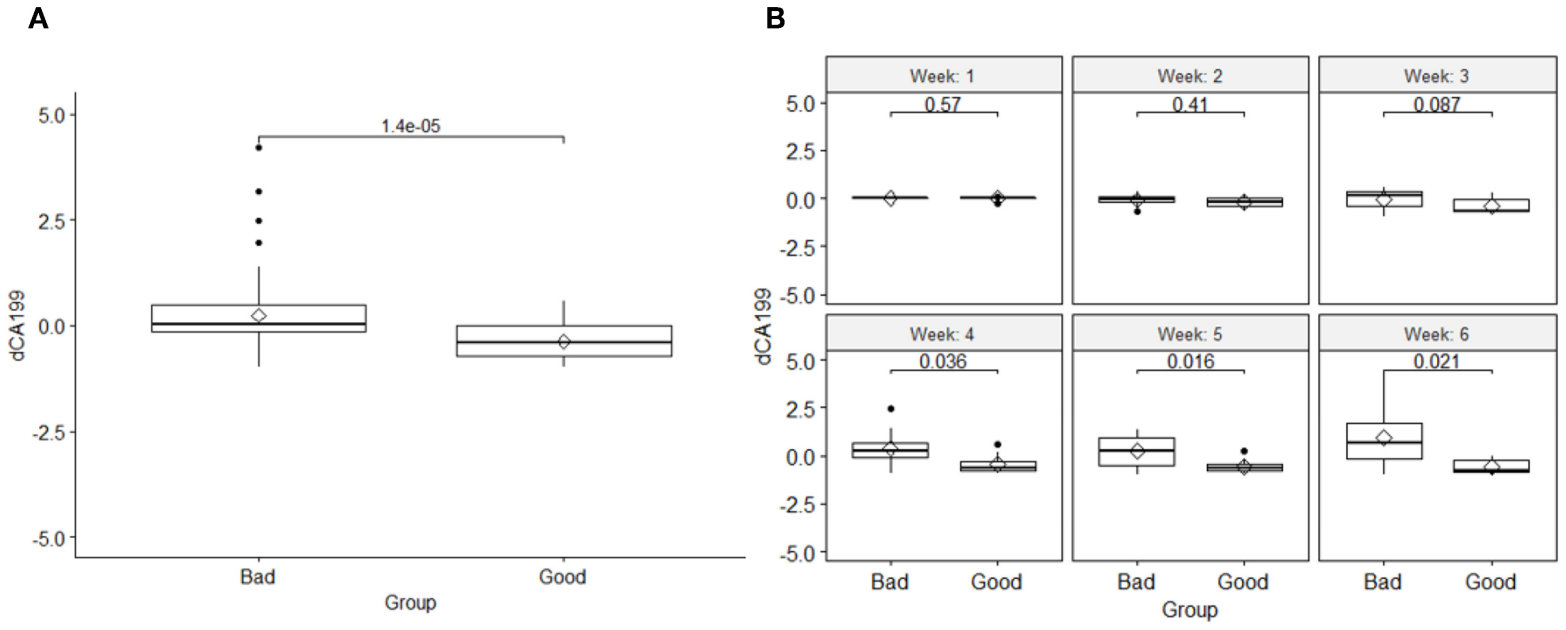
**(A)** Comparison of the changes in the normalized CA19-9 levels between the two response groups and the *t*-test *p*-value, and **(B)** a *t*-test comparison of weekly changes of CA19-9 between the good and bad response groups, indicating that their significant changes began at the fourth week of the treatment.

The results also showed that, like the DRFs the relative net changes in normalized CA19-9 levels were significantly different between the two response groups as shown in Figure 4A. However, unlike the DRFs in case of CA19-9, examining the significant differences between the two response groups on a weekly basis, the earliest significant differences were seen by the fourth week of the treatment as demonstrated by the significant *p*-values of the *t*-test shown in Figure 4B.

**Figure 5:**
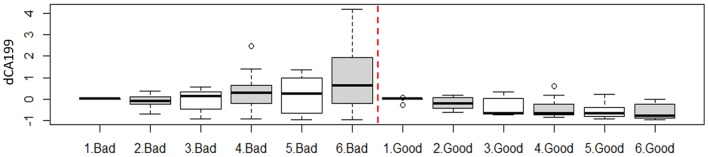
Weekly change of the CA19-9 as function of treatment response. For instance, 1. Bad represent all available fractions within the first week acquired from all patients in the bad response group.

The results also showed that there was an opposite trend between the two response groups utilizing CA19-9 data alone. A relative increase in CA19-9 levels compared to the first fraction during treatment were correlated to a bad response, while a decline in CA19-9 levels, was correlated to a good response. Figure 5 shows the weekly changes of CA19-9 for each response group combing all values for each week for all patients within each response group.

**Figure 6:**
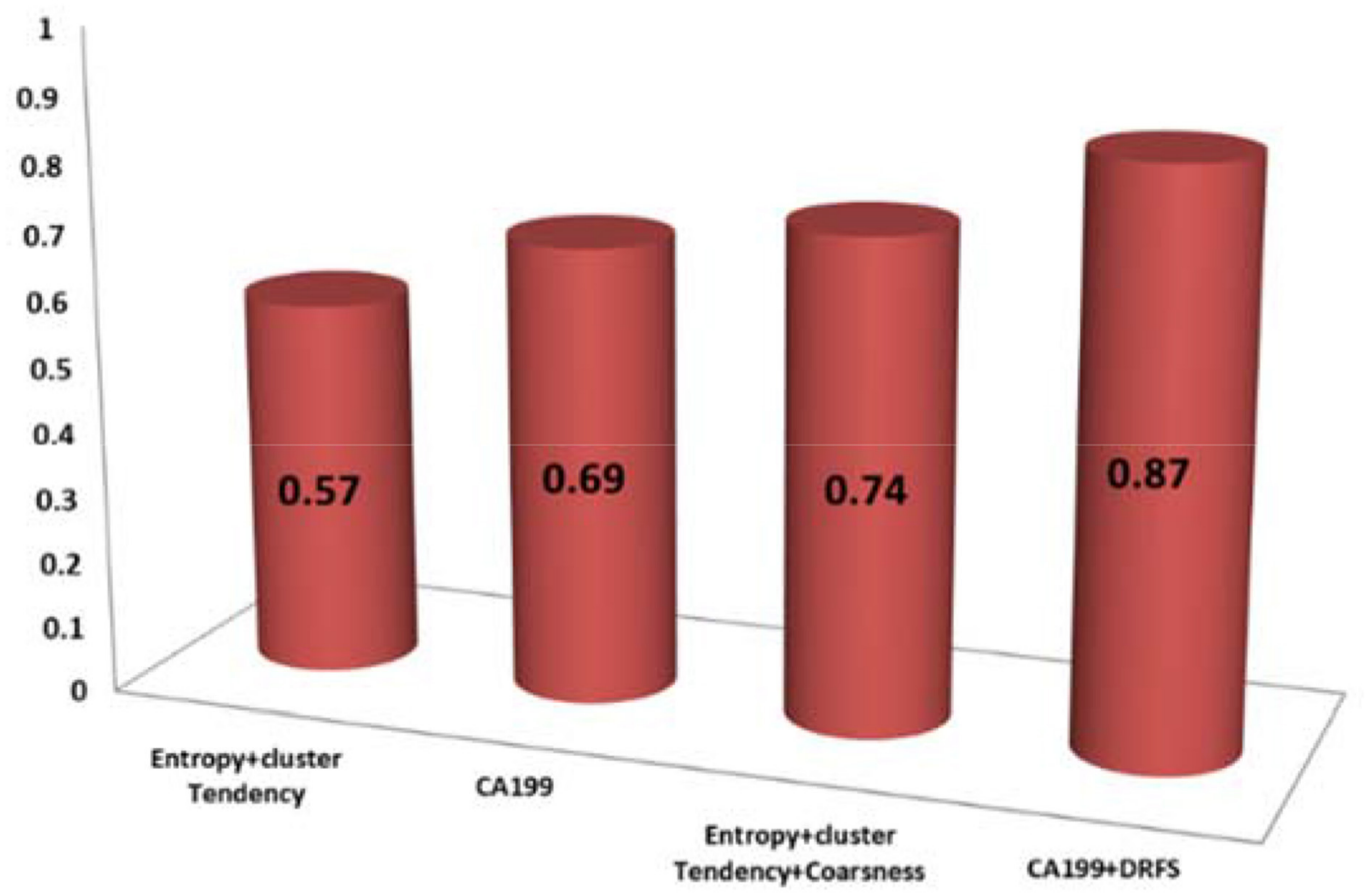
The c-index of the prediction model based on DRFs or CA19-9 alone and their combination.

Of the 13 DRFs correlated to treatment response, three DRFs (entropy, coarseness, and cluster tendency) were statistically relevant and correlated to the changes in CA19-9 with Spearman correlations of (0.65, 0.61, and 0.72, respectively. Incorporating the changes in DRFs to the clinical biomarker CA19-9 enhanced the prediction model as shown by the increase in the concordance statistic of our model from 0.69 using CA-19-9 alone to 0.87 combining CA19-9 to the three DRFs of interest, indicating a stronger power to predict treatment response as shown in Figure 6.

**Figure 7:**
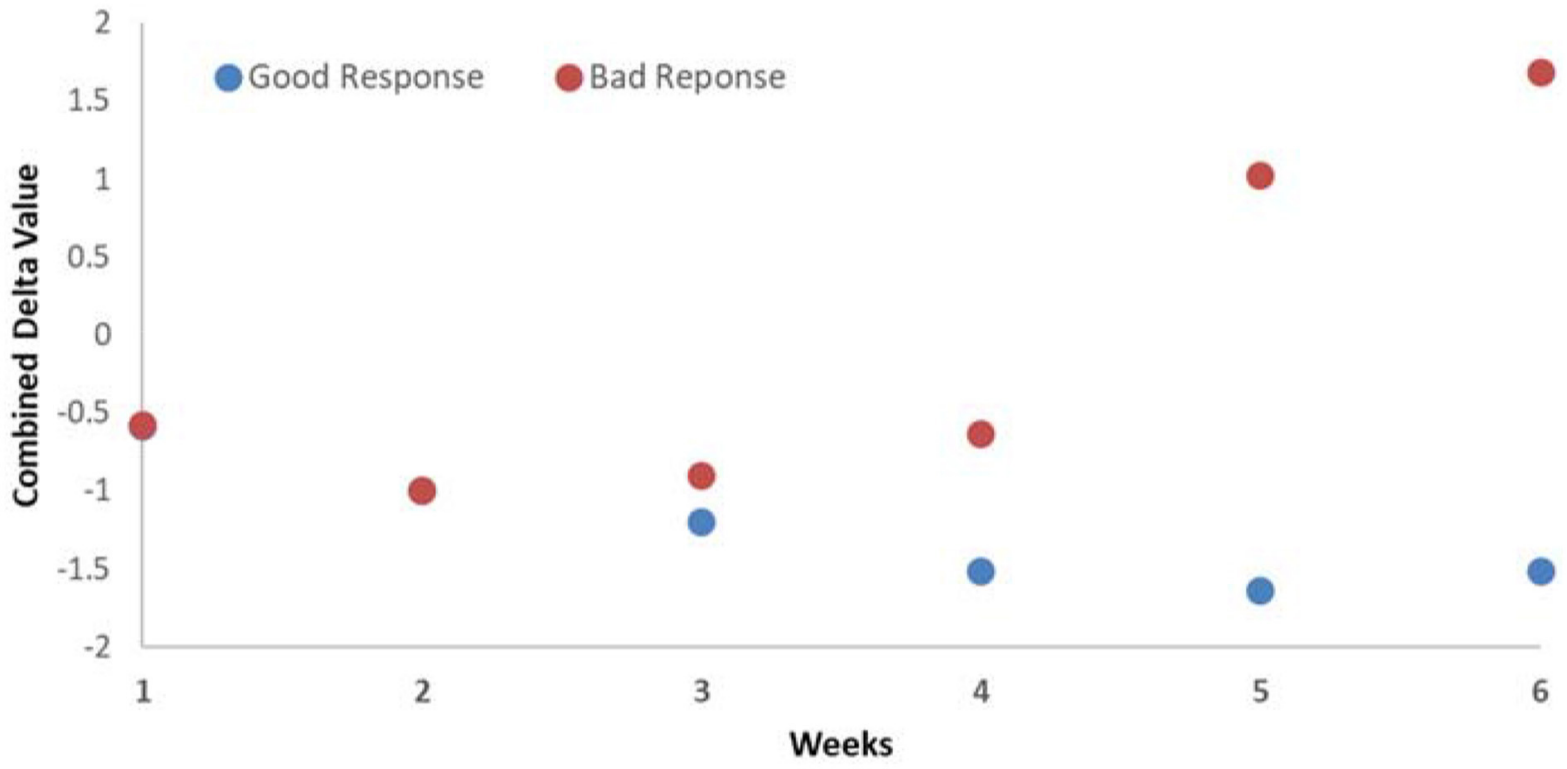
Average weekly changes of the combined biomarker (CA19-9 and DRFs) showing that the detection of good and bad responders begins at the third week during the treatment.

The prediction of good and bad responder was also earlier by combining the changes in the DRFs to CA19-9. The prediction power changed from the fourth week of treatment with CA19-9 alone to the third week combining CA19-9 and DRFs, as shown in Figure 7. Such early detection of treatment response would allow more time to adapt the treatment if necessary.

**Figure 8:**
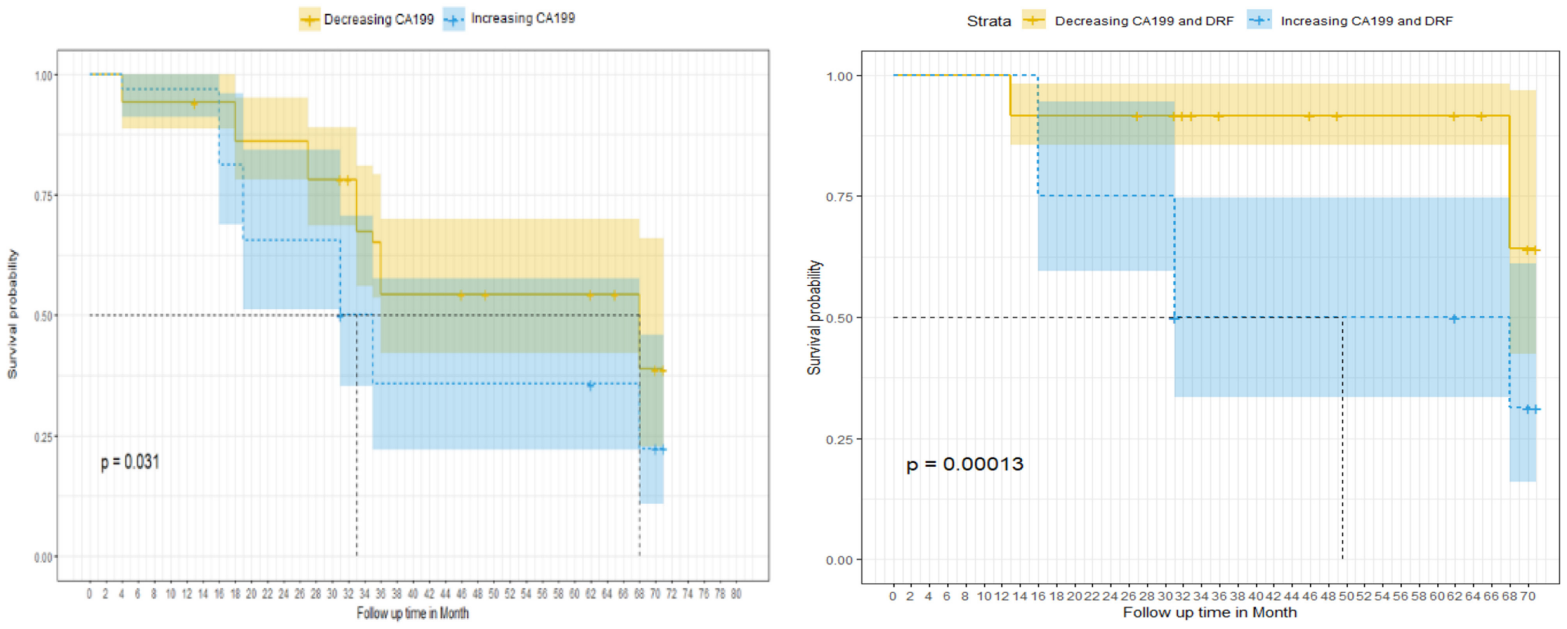
Survival curve as a function of the normalized CA19-9 changes between the increasing and decreasing CA19-9 groups without **(left)** and with **(right)** adding DRFs.

For survival correlations, the univariate analysis showed that patients with decreasing CA19-9 had an improved median survival (68 months) compared to those with increasing levels (33 months). The 5-years survival was improved for the decreasing CA19-9 group (58%) compared to the increasing group (36%). The survival probability was enhanced by combining DRFs to CA19-9 with an improved median survival in the increasing group (49 months) and 68 months in the decreasing group. The 5-years survival probability was also improved to 93% in the decreasing CA19-9 with DRF group compared to the increasing group (50%). Figure 8 show the survival curve comparing group with increasing CA19-9 vs. those with normalized or decreased CA19-9 during treatment (right) and increased vs. decreased survival curves after combining DRFs to CA19-9 (left).

The Cox proportional multivariate hazard analysis showed that a treatment related decrease in CA19-9 levels (*p* = 0.031) and DRFs (*p* = 0.001) were predictors of survival. Table 1 summarize the hazard ratio and the 95% confidence intervals with the corresponding *p*-value. The hazard ratio was reduced from 0.73, *p* = 0.032 using CA19-9 only to 0.58, *p* = 0.028 combining DRFs with CA19-9.

**Table 1:** Hazard ratio and the 95% confidence interval with *p*-values.

**Variate**	**HR (95% CI)**	**Coefficient (*P*)**
Sex	1.3 (0.48–3.4)	0.63
Age	1.7 (1.9–14)	0.31
Response	1.5 (0.67–1.53)	5.40E-05
Cluster tendency	1.1 (0.11–1.3)	0.001
Entropy	0.96 (0.22–1.7)	0.008
Coarseness	0.83 (0.4–1.3)	0.04
CA19-9 + Cluster tendency	0.78 (0.48–1.9)	0.001
CA19-9 + Entropy	0.77 (0.07–1.3)	0.005
CA19-9 + Coarseness	0.76 (0.15–0.79)	0.0007
3 DRFs	0.75 (0.1–1.1)	0.006
CA19-9	0.73 (0.12–0.8)	0.032
CA19-9 + 2 DRFs	0.68 (0.09–1.07)	0.001
CA19-9 + 3 DRFs	0.58 (0.14–1.16)	0.028

## Conclusions

The combination of CT delta radiomics and the clinical biomarker CA19-9 leads to improved prediction of treatment responses for CRT of pancreatic cancer, as compared to radiomics or CA19-9 alone. The combined biomarkers could predict treatment response sooner, during the treatment, increasing the possibility for response-based treatment adaptation, thus, improving treatment outcomes.

## Discussion

As demonstrated in this work, combining the clinical biomarker CA19-9 and delta-radiomics can increase their prognostic value as compared to using either of them alone and increase the confidence in the prediction. CA19-9 is a promising clinical biomarker, however its use can be limited due to the false positive rate caused by certain co-morbid conditions (7–10). Thus, it was important to find another biomarker that confirms the change in CA19-9 is a tumor related response, increasing the sensitivity of CA19-9 and reducing its false positive rates.

Radiomics analysis has been associated with several clinical end points, several researchers showed that it has a potential to be developed into an imaging biomarker using different imaging modalities. We showed earlier that CT based radiomics has a potential in a variety of applications (11–19). Researchers also showed that it has promise using MR images for instance, Wibmer et al. (33) and Vignti et al. (34) showed the radiomics features as potential imaging biomarkers in prostate cancer. Diehn et al. (35) showed that image features extracted from MRI can predict global gene expression patterns in patients with glioblastoma multiforme. Gilles et al. (36) show the potential of radiomics to enable better-biopsy informed decisions in patients with prostate, bladder, and metastatic breast cancer. However, challenges are associated with the use of radiomics due to the restriction for using the data acquired with technical (e.g., different scanners or different protocols) and patient (e.g., respiration motion, stent artifacts) variations. Thus, it's important to account for any technical variations prior to feature extraction, to assure the reproducibility and the repeatability (19). The presence of apparent artifacts can affect the textures extracted and hence, reduce the prognostic value of radiomic features. Thus, caution needs to be used to avoid slices with apparent artifacts before extracting the radiomic features. In this study, we limited our analysis to the patients scanned on the same CT scanner and imaging protocol and we used DRFs that were not prone to those variations (19).

Usually CA19-9 comparisons are made retrospectively between pre- and post-operative values to assess treatment response. However, this does not allow treatment response prediction during treatment and early intervention for online adaptation. In this retrospective study, three DRFs were found to be correlated to CA19-9 and to enhance its prognostic value and hence, the usefulness of the clinical biomarker for treatment response assessment. Even though we included the most possible homogeneous data set in our analysis, non-tumor related changes during treatment (i.e., inflammation or fibrosis) can elevate CA19-9. DRFs, however, can rule out such changes. In this study for outcome predictions we used the pathological treatment response as our outcome and used DRFs that showed significant differences during treatment and correlate to relative net change of CA19-9 to test the improvement in outcome prediction based on both. The correlation between the CA19-9 and DRFs demonstrates the value of adding delta radiomics to the clinical biomarker. Combining CA19-9 and DRFs leads to a lower hazard ratio and earlier treatment response prediction as compared with CA19-9 or DRF alone.

Given these initial promising results, a future prospective study will be designed to collect CA19-9 on a weekly basis with a daily collection focusing on the third and fourth week of treatment. In this analysis, the number of patients with additional CA19-9 were limited as most pancreatic cancer patients only have pre and post-CA19-9 test results. Certainly, these results need to be fully validated and thoroughly tested with larger patient datasets. Future studies can also include examining the effect of different chemo-agents in chemo-only neoadjuvant treatments on treatment response as correlated to the CA19-9 and DRFs extracted using multimodality images. Precise oncologic profiling of the tumor using combination of CA19-9 and DRFs can lead to an early prediction of treatment outcome, allowing for treatment adaptation for patient specific treatment. With these further studies, the combination of CA19-9 and DRFs may be developed as invaluable tools for adaptive radiation therapy.

## Data Availability Statement

Data and codes that support the finding of this study are available from the corresponding author upon reasonable request.

## Ethics Statement

Retrospective, Medical college of Wisconsin IRB approved HIPPA compliant study (consent forms are waived for retrospective study).

## Author Contributions

HN and XL conceived the idea and designed the study. HN compiled and analyzed the patient data, developed the process, and drafted the manuscript. XL supervised the data analysis, edited, and finalized the manuscript. CZ verified the statistical analyses, helped interpreting the results, and reviewed the manuscript. BE, WH, ST, and LW collected the patient data, verified the ROI contours, and edited the manuscript.

### Conflict of Interest

The authors declare that the research was conducted in the absence of any commercial or financial relationships that could be construed as a potential conflict of interest.
